# Regional Differences in Mucociliary Clearance in the Upper and Lower Airways

**DOI:** 10.3389/fphys.2022.842592

**Published:** 2022-03-09

**Authors:** Troy D. Rogers, Brian Button, Samir N. P. Kelada, Lawrence E. Ostrowski, Alessandra Livraghi-Butrico, Mark I. Gutay, Charles R. Esther, Barbara R. Grubb

**Affiliations:** ^1^Marsico Lung Institute, University of North Carolina School of Medicine, Chapel Hill, NC, United States; ^2^Department of Genetics, University of North Carolina at Chapel Hill, Chapel Hill, NC, United States

**Keywords:** mucociliary clearance, mucus blanket, tracheal posterior membrane, posterior nasopharynx, tracheal walls

## Abstract

As the nasal cavity is the portal of entry for inspired air in mammals, this region is exposed to the highest concentration of inhaled particulate matter and pathogens, which must be removed to keep the lower airways sterile. Thus, one might expect vigorous removal of these substances via mucociliary clearance (MCC) in this region. We have investigated the rate of MCC in the murine nasal cavity compared to the more distal airways (trachea). The rate of MCC in the nasal cavity (posterior nasopharynx, PNP) was ∼3–4× greater than on the tracheal wall. This appeared to be due to a more abundant population of ciliated cells in the nasal cavity (∼80%) compared to the more sparsely ciliated trachea (∼40%). Interestingly, the tracheal ventral wall exhibited a significantly lower rate of MCC than the tracheal posterior membrane. The trachealis muscle underlying the ciliated epithelium on the posterior membrane appeared to control the surface architecture and likely in part the rate of MCC in this tracheal region. In one of our mouse models (*Bpifb1* KO) exhibiting a 3-fold increase in MUC5B protein in lavage fluid, MCC particle transport on the tracheal walls was severely compromised, yet normal MCC occurred on the tracheal posterior membrane. While a blanket of mucus covered the surface of both the PNP and trachea, this mucus appeared to be transported as a blanket by MCC only in the PNP. In contrast, particles appeared to be transported as discrete patches or streams of mucus in the trachea. In addition, particle transport in the PNP was fairly linear, in contrast transport of particles in the trachea often followed a more non-linear route. The thick, viscoelastic mucus blanket that covered the PNP, which exhibited ∼10-fold greater mass of mucus than did the blanket covering the surface of the trachea, could be transported over large areas completely devoid of cells (made by a breach in the epithelial layer). In contrast, particles could not be transported over even a small epithelial breach in the trachea. The thick mucus blanket in the PNP likely aids in particle transport over the non-ciliated olfactory cells in the nasal cavity and likely contributes to humidification and more efficient particle trapping in this upper airway region.

## Introduction

The nasal cavity provides the first line of defense in clearing inhaled air of particulate matter and pathogens that could affect the sterility and health of the lower airways. Indeed, the nasal cavity has been described as the “scrubbing tower” for the lower airways (Brain, 1970). Without the protection provided by the nasal cavity, the lower airways would be exposed to a much higher burden of harmful toxins and pathogens that could ultimately compromise air/blood gas exchange. Although mucociliary clearance (MCC) plays a critical role in maintaining health throughout the respiratory system, one might expect a more robust MCC in the nasal cavity compared to that of the lower airways. An enhanced MCC could be accomplished in several ways: (1) an increase in density of ciliated cells, (2) an elevated rate of ciliary beat frequency, both of which have been shown to increase the rate of MCC (Smith et al., 2008; Sears et al., 2015; Xu and Jiang, 2015; Chatelin and Poncet, 2016; Leung et al., 2019), and (3) an “optimized” mucus layer (e.g., more favorable composition or concentration of mucins) that is more efficient at trapping deleterious inhaled substances and/or is more effectively cleared by the cilia.

The purpose of this study was to compare the rates of MCC in the murine upper airways (nasal cavity) with those of the lower airways (trachea) to determine if a regional difference in the rate of MCC exists and if so, why. Also, we explored the decades old debate as to whether the mucus secretions in these two regions form a continuous blanket (Lucus and Douglas, 1934) or a discontinuous layer over the epithelia (Morgan et al., 1984). While the main focus of our study was on the murine airways, we also compared some of the findings from the murine trachea with results from the rabbit trachea. Our results demonstrate that the rate of MCC in the murine nasal cavity is significantly (∼3–4×) greater than that in the trachea and this increase likely reflects differences in density of ciliated cells, ciliary beat frequency, and mucus volume between the two regions. We also provide evidence that mucus secretions likely exist as a continuous layer covering the airways epithelia in both regions.

## Materials and Methods

All mouse and rabbit studies were approved by the University of North Carolina Institutional Animal Care and Use Committee and performed in accordance with the guidelines and regulations governing the use of these laboratory animals. *C57/BL1/6N* mice, males and females studied at 8–10 weeks of age, were allowed food and water *ad libitum* until the time of euthanasia. *Bpifb1* knockout mice were obtained from the Knockout Mouse Project as previously described (Donoghue et al., 2017). Heterozygous breeding pairs were used to generate homozygous wildtype (WT) and knockout (KO) mice. New Zealand white rabbits (male and female) were studied at >8 weeks of age and allowed food and water *ad libitum* until the time of study. All animals studied were bred and raised at UNC.

### Mucociliary Clearance Measurement

The rate of MCC was measured in the intact nasal cavity [posterior nasopharynx (PNP), nasal region dorsal to the soft palate] as previously described (Ostrowski et al., 2010; Supplementary Figure 1). Briefly, a 35-gauge fused silica cannula (WPI, Sarasota, FL, United States) was used to deposit dry fluorescent beads (3 μm, Polyscience, Warrington, PA, United States) in the anterior nasal cavity of a mouse euthanized ∼2 min previously. The movement of the fluorescent beads through the intact PNP was then visualized under a dissecting scope and recorded. In an attempt to determine if mucus was transported as a continuous blanket or as discontinuous patches or streams, in some preparations, a small incision was made in the PNP (or trachea, see below) and fluorescent beads were directly aerosolized onto the epithelial surface of the preparation for 3–5 s (see Rogers et al., 2018 for a description of aerosolization apparatus), Supplementary Figure 2. The temperature of all preparations was closely monitored using a temperature probe (T Type insect probe, Physitemp Inst. Clifton, NJ, United States) placed alongside the trachea or posterior nasopharynx. The output from the temperature probe was displayed digitally on a Physitemp TCAT-2ac Controller and a small ceramic heater (Wuhostam, China) positioned close to the preparation was used to maintain the temperature of the preparation at the desired level (usually 37^°^C maintained within ±0.1^°^C).

Mucociliary clearance was measured in the intact trachea as previously described (Rogers et al., 2018). Briefly, this was done by a 5-min aerosolization of 200 nm fluorescent beads (Vitrogen, Eugene, OR, United States) into the nares of an anesthetized (3% isoflurane) mouse breathing normally though its nose. The mouse was then quickly euthanized by severing the abdominal aorta, the muscles covering the trachea were removed and the fluorescent beads were tracked on the ventral wall through the closed, intact trachea (Rogers et al., 2018) (It should be noted that the 200 nm beads were too small to be individually visualized by our system. However, the beads quickly clump into larger groups of particles on the airway surface which are easily visualized and tracked). The smaller beads were used for tracheal MCC as they more readily passed through the mesh of our aerosol generator. We found no effect of particle size on the rate of MCC (Rogers et al., 2018). To measure MCC on the tracheal posterior membrane, a small incision was made in the ventral tracheal wall and the beads on the posterior membrane were visualized through this incision. The opened preparations were continually humidified by placing the output from a HM500 ultrasonic humidifier within 5 cm of the preparation so that a cloud of humidity continually enveloped the preparation. In all studies the mice were euthanized by severing the abdominal aorta while on a surgical anesthetic plane induced with 3% isoflurane. In all studies different mice were used for the tracheal and PNP measurements.

For validation of concepts, MCC was also measured on the posterior membrane in a small group of rabbits. See Supplementary Methods.

### Histology

Tissues for light microscopic analysis were fixed in 10% neutral buffered formalin, embedded in paraffin, and thin sections were cut and processed for staining with H&E, AB, PAS, or Richardson’s stain. For electron microscopy, tracheas and tissue from posterior nasopharynx were carefully removed, and processed as previously (Grubb et al., 2007). Sections for cilial length determination were stained with H&E and examined at 40× magnification.

### Cell Counting

The % ciliated cells lining the epithelial surface of the trachea and nasopharynx were determined by counting the total number of ciliated cells in high-magnification micrographs of histological cross sections of the trachea or nasopharynx. This number was divided by the total number of cells counted along the basement membrane of each respective tissue type and multiplied by 100. Approximately 200 total cells were counted in nasopharynx or the trachea from each mouse.

### Measurement of Ciliary Beat Frequency

Ciliary beat frequency (CBF) was measured *in situ* in the upper airways (intact nasopharynx) or lower airways (intact trachea) of the mouse immediately after euthanasia. Most of the published studies on CBF involve removal of the tissue from the animal or study CBF in cultured tissue. In contrast, the *in situ* method described herein may more closely resemble the *in vivo* situation, as the region of interest is not opened to ambient air, thus preserving humidity and the native airway surface milieu. The nasopharyngeal region was prepared for CBF measurement as previously described for mucociliary clearance measurements (Ostrowski et al., 2010). A thin layer of water-equilibrated mineral oil was layered over the intact ventral surface of the exposed intact PNP and covered with plastic wrap to prevent evaporation (see Supplementary Figures 1A,B). A similar procedure was used for tracheal preparations. To measure CBF, these preparations were placed immediately after euthanization under a dissecting scope (10× magnification) outfitted with a digital camera (Basler, Germany) interfaced to a computer using Basler image acquisition software. The preparation was lighted with a DC high intensity red LED (655 nm); Luxeon making light reflected from the beating cilia easily seen. Temperature was maintained as described for MCC measurements. To minimize vibrations that interfered with CBF measurements, the scope was placed on an air table (TMC, Peabody, MA, United States). For both preparations, it took approximately 3 min from the time of euthanasia until the preparation was under the scope and data acquisition commenced. CBF was measured for a 2 s period at 10, 15, 20, and 25 min after euthanasia. The data were collected at 100 frames/s and analyzed by Sisson-Ammons Video Analysis (SAVA) software as previously described (Sisson et al., 2003; Sears et al., 2015). To validate our *in situ* CBF method, we first studied CBF in both nasopharyngeal and tracheal preparations as they were cooled from ∼37 to 10^°^C. We observed that both preparations exhibited a fairly linear decrease in CBF with temperature (Supplementary Figure 3) as previously reported (Sears et al., 2015).

### Quantitative Comparison of Mucus in Nose vs. Trachea of the Mouse

To quantitatively compare the amount of secreted mucus in the posterior nasopharynx to the trachea, we used the recently published method of Esther et al. (2017) comparing the mass of sialic acid (a surrogate marker for mucins) measured by mass spectrometry. To collect samples, we opened the region of interest and gently placed a small nitrocellulose filter (5 mm × 0.8 mm cut to size using a CO_2_ laser) on the apical surface of the tissue (dorsal wall of posterior nasopharynx, lateral wall or posterior membrane of the trachea). The paper strips were left in place for 3 min during which time the opened region was covered with moistened paper and plastic wrap to preserve humidity. The sialic acid and urea concentrations were measured on samples eluted from the filter paper and serum urea was measured from a blood sample drawn immediately after euthanasia as previously described by Esther et al. (2017). We corrected recovery of samples on the filter paper strip by comparing urea on the filter paper to serum urea (filter paper sialic acid × serum urea)/filter paper urea and the corrected sialic acid concentrations are reported.

### Statistics

All data are shown as means ± SEM. When comparing two groups, a student’s *t*-test was used. If more than two groups were compared, a one-way ANOVA was used; a two-way ANOVA was used to analyze CBF data. A *p*-value of <0.05 was considered statistically significant.

## Results

### Rate of MCC in the Posterior Nasopharynx and in the Trachea

**FIGURE 1:**
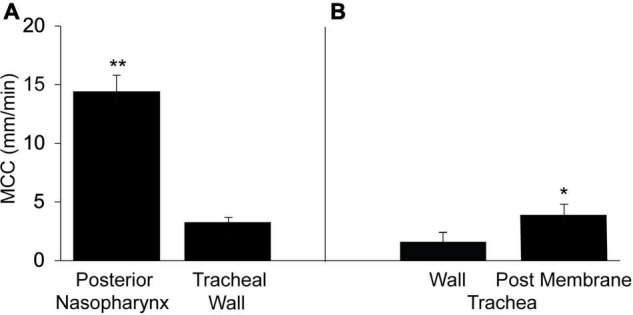
Mucociliary clearancein the murine posterior nasopharynx (PNP) and trachea (wall region subtended by cartilage) and posterior membrane (non-cartilaginous regions subtended by trachealis muscle). **(A)** Data from intact preparations and **(B)** data from open preparations. Data are shown as means ± SEM. *N* = 10 mice for PNP and *n* = 8 mice for both tracheal wall and posterior membrane. **p ≤ 0.001 MCC in PNP compared to tracheal wall or tracheal posterior membrane. *p ≤ 0.05 tracheal wall vs tracheal posterior membrane.

To compare the rate of MCC in the upper airways (nasal cavity-posterior nasopharynx) with that of the lower airways (trachea), we determined the rate of MCC in the intact murine posterior nasopharynx *in situ* to be ∼14.4 ± 1.4 mm/min (Figure 1A), in agreement to our earlier studies (Grubb et al., 2016; Chen et al., 2018; Yin et al., 2019). The rate of MCC measured on the intact ventral tracheal wall was significantly less than that measured in the intact nasopharynx, 3.28 ± 0.39 (Figure 1A).

In contrast to the PNP where the architecture is uniform around the circumference of the region, this is not the case for the trachea. The trachea can be divided into two distinct regions: (1) the walls (ventral and lateral) and (2) the posterior membrane (the dorsal wall). While both tracheal regions are populated with ciliated and club cells, the ventral and lateral walls are found only in the cartilaginous region of the trachea. The tracheal cartilage form an incomplete ring and the discontinuous ends of each ring are connected by the trachealis muscle, thus forming the tracheal posterior membrane (Miller, 1913; Grubb et al., 2016). The posterior membrane, attached to the trachealis muscle is covered with an epithelial layer similar to the ventral and lateral walls of the tracheas. The tracheal walls were subjected to minimal perturbation during dissection and most easily imaged allowing measurements of MCC in the intact trachea. However, due to its anatomical location, it was not possible to measure MCC on the posterior membrane of the intact trachea by our technique. However, when the trachea was opened along the ventral surface and MCC measured on the posterior membrane, the rate of MCC was found to be significantly greater on the tracheal posterior membrane than on the ventral tracheal wall (Figure 1B) but still significantly less than the MCC measured in the PNP. The rate of MCC that we measured for the walls of the opened tracheal preparations, was slightly less than we measured in the intact preparations. Therefore the results from the wall of the opened preparations (Figure 1B) are compared to the results for the posterior membrane (also an opened preparation).

### Characteristics of MCC in the PNP and the Trachea

**FIGURE 2:**
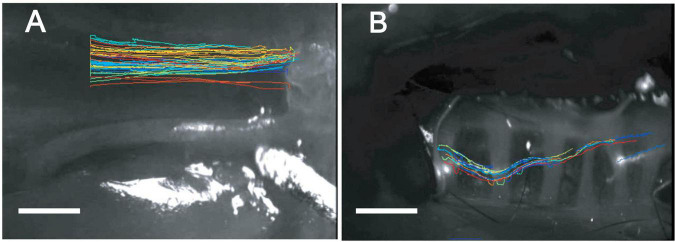
“Spaghetti” plots from representative tissue **(A)** nasopharynx and **(B)** trachea (ventral wall) demonstrating linear tracks of particles in nasopharynx and non-linear tracks of particles in trachea. While individual bead transport cannot be clearly seen in these plots, the over-all pattern of bead transport between the posterior nasopharynx **(A)** and trachea **(B)** is very different between the two regions. Data were analyzed and spaghetti plots generated using ImageJ software with TrackMate.

Visualization of particle transport revealed differences in transport patterns between the upper and lower airway. In the intact PNP, the beads distributed throughout the PNP, and viewed through the basolateral side of the ciliated epithelial layer appeared to all be transported at about the same rate and to coalesce into discrete rafts of beads (Supplementary Video 1). To assess whether formation of the mucus/bead rafts was a property of MCC in the PNP or a phenomenon that occurred when beads were introduced via the anterior nasal cavity, beads were aerosolized directly on the ciliated cells of the opened PNP. In these experiments, the beads did not form large rafts of beads and moved linearly toward the epiglottis at approximately the same rate likely on a blanket of mucus covering the ciliated epithelia (Supplementary Video 2). When direction of transport was analyzed in the intact PNP preparation, the beads appeared to follow linear paths to the oropharynx, Figure 2A.

Visualization of particle transport on tracheal walls revealed that beads appeared to move as streams or rafts of beads rather than on uniform blanket of mucus (Supplementary Video 3), similar to what we have previously reported (Rogers et al., 2018). The rafts or streams of beads appeared to flow cephalad, but often were not linear or parallel to the longitudinal axis of the trachea Figure 2B. In addition, beads were often seen to be transported in discrete rafts/continuous streams moving at different rates. As can be seen in the Supplementary Video 3, significant numbers of non-motile adherent groups of beads were commonly observed in the mouse tracheal preparation as described previously (Rogers et al., 2018) (These adherent beads were never observed in the PNP of a normal WT mouse. The non-motile beads were not included in the rate analysis). Transport of beads on the tracheal posterior membrane was visualized via direct aerosolization onto the posterior membrane of an open trachea. In these experiments, the pattern of bead transport on the tracheal posterior membrane appeared different than observed on the wall, with the beads transported linearly more as a continuous stream to the epiglottis (Supplementary Video 4).

To further validate the pattern of MCC on the tracheal wall vs tracheal posterior membrane, we performed similar experiments on the rabbit trachea. The rabbit trachea is anatomically very similar to the mouse trachea in that no submucosal glands are present and the tracheal posterior membrane is anatomically similar to that of the mouse. In addition, the rabbit trachea is much larger than that of the mouse allowing better visualization of both the wall and posterior membrane on the same preparation. The rabbit trachea exhibited the same pattern of transport as seen in the mouse trachea (Supplementary Video 5), with the lateral walls exhibiting a significantly lower rate of MCC than the posterior membrane (Supplementary Figure 4).

**FIGURE 3:**
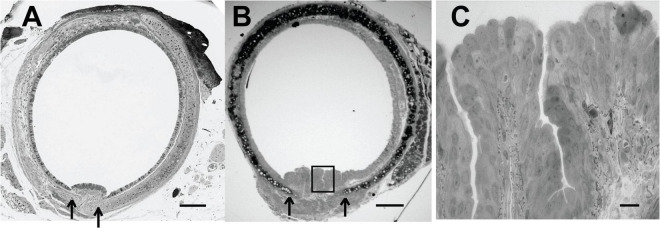
**(A)** Histological section (Richardson’s stain) of a fixed murine trachea exhibiting a contracted trachealis muscle-arrows indicates edge of cartilage. **(B)** Histological section (Richardson’s stain) of a fixed murine trachea exhibiting more relaxation in the trachealis muscle-invaginations of the epithelial layer are evident-arrow at open edges of cartilage. **(C)** Enhanced magnification of posterior membrane from panel **(B)** (boxed region). Scale bar **(A,B)** 200 μm, **(C)**, 20 μm. [Preparations in panels **(A,B)** were from different mice and not processed at the same time and thus the staining intensity is not identical.]

In both the mouse and the rabbit tracheal posterior membrane, the particulate matter (fluorescent beads) appeared to flow in linear streams parallel to the long axis of the trachea. We observed that in fixed murine tracheal sections, when the trachealis muscle was markedly contracted, the posterior membrane was compressed and did not appear to be invaginated (Figure 3A). However, when the trachealis muscle was more relaxed and the cartilaginous rings spread farther apart (Figure 3B), deep troughs appeared in the posterior membrane (Figure 3C). While a relaxed trachealis muscle could create invaginations that carry the observed streams of particles, we were not able to directly assess the contractile state of this muscle in the preparations in which we measured MCC.

**FIGURE 4:**
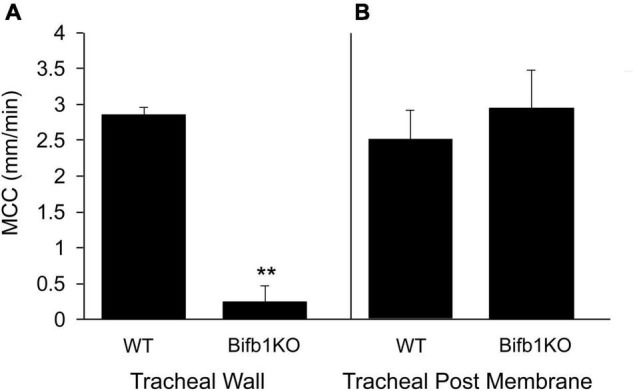
**(A)** MCC on the tracheal wall of the WT or *Bpifb1* KO mouse and **(B)** MCC on the tracheal posterior membrane of the WT and *Bpifb1* KO mouse. *N* = 10 both genotypes for the tracheal wall **p ≤ 0.001 WT wall vs KO wall. *N* = 10 for tracheal posterior membrane (WT) and *n* = 8 posterior membrane KO (In these preparations, it will be noted that the rate of MCC on the posterior membrane is not greater than that on the tracheal wall as we typically observe (Figure 1). This is likely because we measured MCC on the tracheal wall first and we did not open the trachea for posterior membrane measurement until 10–12 min after the wall measurement had been completed. By this time, the MCC on the posterior membrane had slowed slightly (Rogers et al., 2018).

The importance of the posterior membrane in clearing the trachea of particulate matter was demonstrated in a mouse model in which the gene *Bpifb1* was knocked out (Donoghue et al., 2017). The KO mouse exhibited a 3-fold increase in MUC5B protein levels in lung lavage fluid, as well as exhibiting a significant increase in the fraction of intra-luminal airway AB-PAS positive secretions detected histologically (Donoghue et al., 2017), suggesting that the mass/concentration of MUC5B on the tracheal surface was likely increased in *Bpif1* KO mice. In addition, in a more recent paper (Donoghue et al., 2020), it was found that the loss of BPIFB11 resulted in a significant increase in complex viscosity in mucus flakes (i.e., the insoluble portion of BALF airway mucus). Thus, the biophysical properties of mucus are likely changed in this KO strain, resulting in a reduced ability to clear mucus. We found that MCC on the tracheal wall (ventral surface) was markedly reduced in *Bifb1* KO mice compared to *Bpif1* WT mice (Figure 4A; compare Supplementary Videos 6, 7), corroborating previous results (Donoghue et al., 2020) with many of the KO mice exhibiting no bead transport on the ventral wall. Surprisingly, MCC on the tracheal posterior membrane of the KO mouse did not differ from that of the WT mouse (Figure 4B; compare second halves of Supplementary Videos 6, 7). Thus, transport on the tracheal posterior membrane seems more robust than that exhibited by tracheal walls and is effective in clearing particles from the trachea when clearance by the tracheal walls fail. Interestingly, the rate of MCC in the nasopharynx of the *Bpifb1* WT mouse [12.6 ± 1.3 mm/min (*n* = 6)] did not differ significantly from that of the Bpifb1 KO mouse [11.7 ± 1.33 mm/min (*n* = 6)].

### Gross Organization of Mucus on the Surface of Murine Nasopharyngeal and Tracheal Epithelia

**FIGURE 5:**
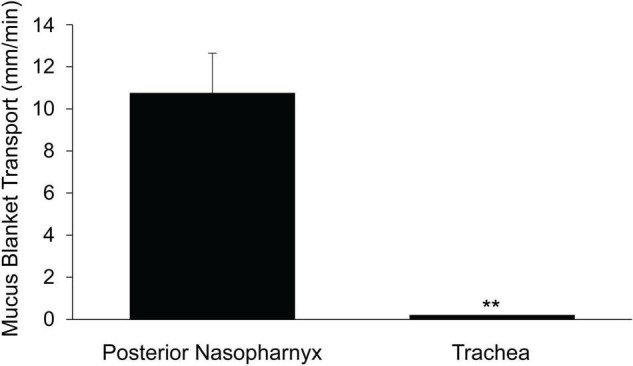
Rate of transport of mucus blanket across region devoid of airway epithelia. *N* = 5 mice for posterior nasopharynx and *n* = 6 mice for trachea. **p ≤ 0.001 mucus blanket transport in PNP vs trachea. The rate of transport of the mucus blanket in a region of the PNP devoid of epithelia (this Figure) did not differ significantly from the rate of bead transport on the PNP intact epithelia (Figure 1).

In the PNP, beads aerosolized directly on the ciliated surface, moved on a uniform blanket of mucus (see Supplementary Video 2). To test the cohesiveness of this mucus blanket, by careful dissection, we created a small breach in the ciliated epithelial layer on the ventral surface of the PNP (instead of the epithelial layer being completely removed as described above) (Supplementary Figure 2). This maneuver exposed an intact blanket of mucus that stretched across the breached (no epithelia) region. As seen in Supplementary Video 8, the mucus blanket overlaying the area devoid of epithelia, was transported at a similar rate to the beads outside of the breach on the ciliated epithelia. The rate of transport of the mucus blanket across the breached region [10.7 ± 1.7 (7), Figure 5] did not differ significantly from the rate of bead transport by the intact ciliated epithelium [14.3 ± 1.4 (8), Figure 1]. To further demonstrate that a cohesive mucus blanket covered the region where the ciliated epithelia were removed, fluorescent beads were aerosolized directly onto the area devoid of epithelia (The aerosol generator was placed where the oral cavity would have been had the lower jaw not been removed). Again, clearly demonstrating the beads’ movement indicated the mucus blanket was transported over the breached epithelial layer similar to particles in the intact PNP (Supplementary Video 9).

A similar protocol was used to determine if a mucus blanket was present on the epithelia covering the ventral wall of the trachea. The epithelial layer was breached by careful dissection of the tissue in the intercartilagenous region, revealing an intact blanket of mucus covering the breach (Supplementary Video 10). However, unlike the PNP, vectorial transport of beads across this breach was not observed (Note that the length of the blanket of mucus in the breached region of the trachea was about 6-fold less than in the PNP (compare scale bars in Supplementary Video 8 vs Supplementary Video 10), yet the beads were not transported over this small breach in the tracheal epithelia). If beads were aerosolized on the outside of the trachea, over the mucus blanket covered by the breach (equivalent to what was done in the PNP), no vectorial transport was observed (Figure 5 and Supplementary Video 10).

### Ciliated Cell Density in Murine Nasopharyngeal and Tracheal Airway Epithelia

**FIGURE 6:**
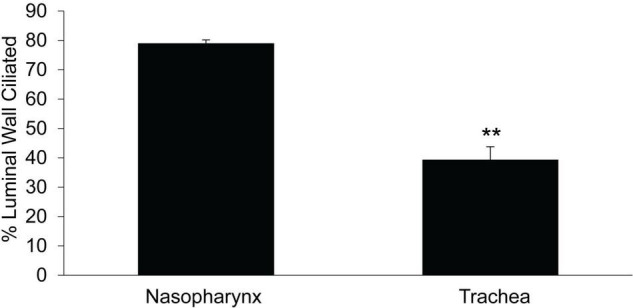
Comparison of % ciliated cells on epithelial layer of the circumference of the trachea vs that in the nasopharynx. Data are from the mid region between the epiglottis and the tracheal bifurcation. PNP *n* = 6 mice, tracheal *n* = 4 mice, **p ≤ 0.01.

**FIGURE 7:**
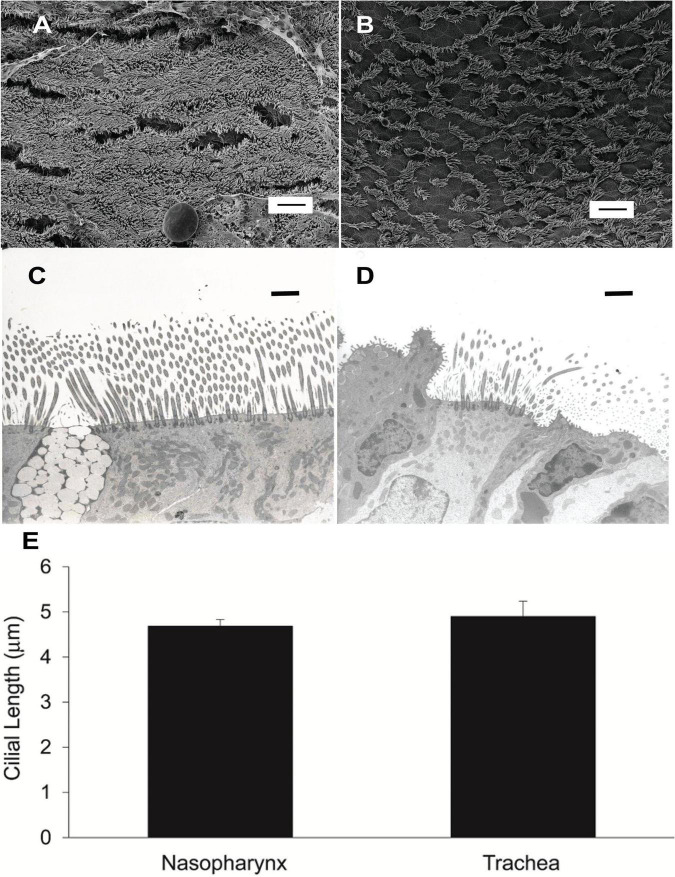
**(A)** Scanning EM image of murine nasopharynx and **(B)** trachea wall demonstrating much more sparse ciliation in the trachea. Scale bare is 10 μm. **(C)** TEM of murine nasopharynx with goblet cell visible between ciliated cells. **(D)** TEM of murine trachea with club cell visible on the left side of image. Scale bars are 20 μm. **(E)** Length of cilia in murine nasopharynx vs trachea (*n* = 6 mice PNP, *n* = 3 mice trachea).

We compared ciliated cell density in the murine PNP to that of the trachea and found that the % ciliated cells per region analyzed was significantly greater in the PNP than in the trachea (Figure 6). We found no difference in the % ciliation on the walls of the trachea (41.9 ± 9) compared to the posterior membrane (37.0 ± 9.7). (The tracheal posterior membranes examined histologically for ciliated cell density were similar to those shown in Figure 3A and did not exhibit the marked groves that can be created due to the contractile state of the trachealis muscle see Figures 3B,C) To examine the ciliated PNP and trachea in more detail, excised preparations were examined by scanning and TEM electron microscopy. A scanning EM image of the ciliated PNP epithelia (Figure 7A) compared to that of the trachea wall (Figure 7B) clearly illustrates the sparse ciliation of the trachea compared to the more abundant ciliation of the PNP. In the PNP, the abundant cilia appeared carpet-like, making it difficult to distinguish individual cells, while in the trachea, individual ciliated cells were clearly identifiable (Figure 7B). Transmission electron micrographs demonstrate that in the PNP, the cilia make contact from cell to cell (Figure 7C), whereas in the trachea, there are clearly gaps between the ciliated cells, mainly occupied by club cells (Figure 7D).

### Cilial Length in Murine Nasopharyngeal and Tracheal Airway Epithelia

To determine if the length of the cilia partially accounted for the increased MCC of the PNP, we measured the length of cilia in the murine PNP and trachea. We found that the length of the cilia did not differ significantly between the PNP and trachea (Figure 7E).

### Cilial Beat Frequency in Murine Nasopharynx and Trachea Airway Epithelia

**FIGURE 8:**
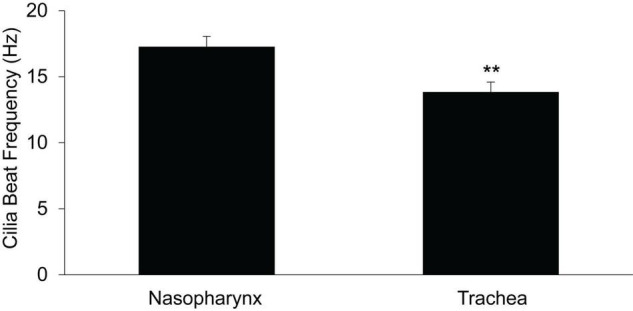
Ciliary beat frequency in nasopharynx vs trachea (*n* = 7 mice, each region) PNP vs trachea. **p ≤ 0.01.

Cilial beat frequency (CBF) was measured in the PNP and trachea to determine if the magnitude of CBF correlated with the rate of MCC. We determined the CBF as a function of time after euthanasia in PNP and trachea maintained at 37^°^C. For both regions, we measured CBF in seven mice at 5-min intervals beginning 10 min post euthanasia. Six to ten measurements were taken for each mouse at each region/time. As there was no significant effect of time (from 10 to 45 min) on the mean rate of CBF for either the nasopharynx or the trachea (two way-ANOVA), the results from each time interval were averaged and the mean of each region is shown in Figure 8. The CBF was slightly, but significantly greater (∼20%) in the PNP, suggesting that this may partially account for the increased MCC in the nasal cavity.

### Mucus Mass in Nasopharynx and Trachea

**FIGURE 9:**
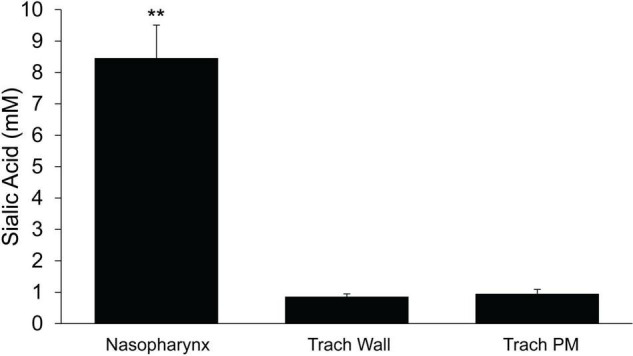
Mass of sialic acid recovered on filter paper strips placed in the nasopharynx or trachea (wall or posterior membrane) *n* = 9 mice/group. ≤ 0.001 PNP vs tracheal wall or tracheal posterior membrane. **p ≤ 0.001.

To determine if the mass of mucus present on the surface differed between the upper and lower airways, we used sialic acid as an index of mucus mass as previously described (Esther et al., 2017). Samples were collected on filter papers and analyzed by mass spectrometry. The absolute mass of sialic acid (corrected for recovery by serum urea, see section “Materials and Methods”) recovered from the PNP was ∼10-fold greater than recovered in the trachea (Figure 9). There was no significant difference in the mass of sialic acid recovered between the walls and the posterior membrane of the trachea (Figure 9).

## Discussion

Mucociliary clearance, effected by the coordinated beating of cilia, is the first line of defense to maintain pulmonary health and is responsible for clearing both the upper (nasal cavity) and lower airways of mucus, particulate matter and pathogens. The absence of effective MCC, in diseases such as primary ciliary dyskinesia and cystic fibrosis, leads to frequent and severe pulmonary infections, causing significant morbidity and mortality (Boat et al., 1989; Donaldson and Boucher, 2003; Rowe et al., 2005; Eden et al., 2019). Since the nasal cavity is the port of entry of ambient air to the respiratory tract, it might be expected that a more vigorous MCC system may be present in the nose where the largest burden of inhaled particulate matter and pathogens would be found. By the time the inspired air has reached the trachea, it has been warmed, humidified and much of the inhaled particulate matter and noxious gases have been removed (Brain, 1970; Williams et al., 1996; Karamaoun et al., 2018).

We have previously reported that MCC in the nasal cavity of WT mice can be measured by tracking the movement of fluorescent beads deposited in the intact nasal cavity and have demonstrated that the rate of MCC measured in the PNP is sensitive to genetic modifications of the MCC system (Ostrowski et al., 2010; Roy et al., 2014). We now report that the rate of MCC in the murine nasal cavity (posterior nasopharynx) was ∼4× fold faster than in the trachea. Numerus factors can influence the rate of MCC, including cilial length, waveform, beat frequency, density, mucus composition, concentration and rheology (Button et al., 2012; Sears et al., 2015; Chatelin and Poncet, 2016; Kikuchi et al., 2017; Mall et al., 2020). In addition, the regulation of ion and fluid transport that regulates the hydration of the airway surface layer, is critical for effective MCC, as illustrated by the severe defects of MCC in cystic fibrosis patients (Boat et al., 1989; Rowe et al., 2005; Button et al., 2012; Boucher, 2019).

With respect to airway hydration and its role in MCC, our prior studies in mice (Grubb et al., 1994, 2001, 2007; Rock et al., 2009; Ostrowski et al., 2010) suggest no major differences in the basal short-circuit current, nor the responses to amiloride or forskolin in the trachea compared to the nasopharynx. However the tracheal response to UTP (TMEM 16A response) is markedly enhanced compared to that in the PNP (Grubb et al., 1994, 2001, 2007; Rock et al., 2009; Ostrowski et al., 2010), suggesting that the tracheal epithelia likely have an enhanced ability to secrete liquid as a result of nucleotide stimulation compared to the surface epithelial of the PNP. Since the nasal cavity is responsible for humidifying the inspired air, secretions from the numerous submucosal glands (absent in all but the uppermost murine trachea), likely contribute to the overall liquid homeostasis of this region.

In our study, as cilial length did not differ significantly between the two regions, thus we have eliminated it from the list of potential sources of MCC differences in the two regions. CBF was found to be significantly greater (∼20%) in the PNP compared to the trachea, but prior studies suggest this change would only increase MCC by 15–20% (Sears et al., 2015), not nearly enough to account for the difference in the rate of MCC we routinely measured in the two regions.

One of the easiest factors to reconcile the difference in the rate of MCC between the two regions is ciliary density. In the mouse, the nasal epithelium (posterior nasopharynx) has about twice the density of ciliated cells (∼80% ciliation) as has the trachea (∼40% ciliation). There are numerous reports in the literature documenting that the murine trachea is very sparsely ciliated [30–37% (Pack et al., 1980; Klein et al., 2009; Kiyota et al., 2014)] and that ciliated cells in the trachea are found in scattered patches (Pack et al., 1980). In fact, Pack et al. (1980) commented that in the murine trachea the ciliated cells “were so far apart that is difficult to envisage how such a mucous sheet would be propelled.” As it has been previously shown that there is a positive correlation between cilial density and the rate of MCC (Smith et al., 2008; Xu and Jiang, 2015; Chatelin and Poncet, 2016; Leung et al., 2019), it is likely that the difference in the rate of MCC between the PNP and trachea is in large part a result of significantly more abundant ciliated cells in the PNP. Additionally, this likely explains the circuitous route (non-linear) that beads/mucus follows during MCC in the trachea, compared to the linear paths observed in the PNP. However, especially with respect to the trachea, the number of ciliated cells per basement membrane is not the only determinant of MCC because the MCC rate is significantly greater on the tracheal posterior membrane than the tracheal wall, yet the number of ciliated cells did not appear to differ between these anatomic regions in the mouse (see section “Discussion”).

Our study also helps to address the long standing controversy over whether the surface of airways is covered with a continuous mucus blanket (Iravani and As Van, 1972; Sturgess, 1977; Morgan et al., 1984) with some reporting a continuous mucus blanket covering the lower airways (Sturgess, 1977) and nasal cavity (Morgan et al., 1984) and others reporting no mucus blanket on the lower airways (Iravani and As Van, 1972). Others have noted the mucus covering the lower airways of rats and mice is non-homogenous in composition and thickness (Sims and Horne, 1997; Ehre et al., 2012; Fakih et al., 2020). Iravani and Melville (1975) have reported “flakes” or continuous rivers of mucus streams being transported in lower airway preparations in a variety of species, yet they reported no “blanket” of mucus being present. In a recent paper Fakih et al. (2020) reported that in an *ex vivo* murine tracheal preparation, mucus was transported as discontinuous “clouds” on the tracheal surface, which appeared to be devoid of a blanket of mucus. In the present study we have demonstrated that both the PNP and the tracheal epithelia appear to be covered with a cohesive visco-elastic layer, which we term a “mucus blanket.” While we did not directly measure the composition of this mucus blanket, it is likely different in the nasal cavity compared to the trachea. Mucus in the nasal cavity is composed of both MUC5B and MUC5AC and probably other mucins and proteins secreted by the various glands present in this tissue (Roy et al., 2014). In the lower airways, the prominent source of mucus are the club cells in the surface epithelium and MUC5B is the main secreted mucin (Evans et al., 2004; Young et al., 2007; Davis and Dickey, 2008; Zhu et al., 2008; Fahy and Dickey, 2010; Okuda et al., 2019; Fakih et al., 2020). Thus, we have demonstrated that in the PNP particles are cleared on a moving mucus blanket that can be pulled over large areas completely devoid of cilia (This is likely the mechanism of mucus transport over the olfactory epithelia which are devoid of motile cilia). In contrast in the trachea, the visco-elastic blanket does not appear to move, and particles are cleared in rafts or streams of mucus, similar to what has been demonstrated in the isolated murine trachea (Fakih et al., 2020).

It was interesting that the murine trachea walls did not appear to effectively transport particles directly aerosolized on the tracheal walls (Supplementary Video 4) yet beads aerosolized via nasal inhalation yielded streams or rafts of beads (and presumably mucus) that were transported cephalad (although not necessarily linearly) on the tracheal walls (Supplementary Video 3). It may be that the individual beads aerosolized directly onto the tracheal surface landed on areas too sparsely ciliated for effective MCC. However, the beads nasally inhaled and distally deposited may have induced mucus secretion producing larger aggregates of beads/mucus which than could be cleared. However, even in the normal murine trachea we often observed stationary plaques of beads that failed to be transported. Many others (Brownstein, 1987; Donnelley et al., 2014; Roy et al., 2014; Veres et al., 2017; Rogers et al., 2018) also noted “stagnant” mucus on the walls of lower airway preparations similar to what we have observed in the trachea.

The tracheal posterior membrane in both the mouse and rabbit exhibited a significantly greater rate of MCC than did the tracheal ventral wall. In the mouse, the tracheal posterior membrane was able to transport particles under conditions that were not favorable to wall transport (*Bpifb1* KO mice). Whereas others have reported more ciliation on the posterior membrane in the hamster (Gabridge et al., 1977) and more mucus on the rat’s posterior membrane (based on histological images) (Sims and Horne, 1997), we found neither more cilia [measured when the posterior membrane was more contracted (Figure 3A)], nor a greater mass of mucus on the posterior membrane in the mouse compared to the tracheal walls (Figure 9). Because the posterior membrane is attached to the underlying trachealis smooth muscle, innervated by the parasympathetic nerves, it would be expected that changes in muscle contractility due to cholinergic stimulation would alter the surface architecture of the posterior membrane. Indeed, longitudinal “rugae” formed by changes in contractility of the trachealis muscle have been reported (Miller, 1913). Studies have demonstrated spontaneous contractile activity of the trachealis muscle both *in vivo* and *in vitro* (Widdicombe, 1963; Souhrada and Dickey, 1976) and it was speculated that this activity might have a role in mucus elimination (Souhrada and Dickey, 1976). An early paper reported that, depending on the degree of contraction of the trachealis muscle, the posterior membrane can exert a “squeegee effect” whereby liquid on the tracheal wall is pushed (or pulled) onto the posterior membrane (Coburn, 1972) and from there transported cephalad. While the invaginations themselves are not visible at the magnification used to image the trachea in our MCC studies, these “lines” resulting from an accumulation of beads (especially the rabbit tracheal preparations, Supplementary Video 5) are likely the valleys created by the rugae. Others have shown that there appears to be “streaming” on the posterior membrane and particles tended to move from the tracheal walls to the posterior membrane (Asmundsson and Kilburn, 1970). Similarly, our studies demonstrate that beads aerosolized onto the walls and posterior membrane tend to travel from the tracheal wall toward the posterior membrane and the beads are then carried cephalad in longitudinal lines. When the posterior membrane is overwhelmed with particulate matter (Supplementary Videos 6, 7), these streams are obscured, and the entire tracheal posterior membrane is covered with beads/mucus which are transported cephalad. At this point, we have not investigated the mechanism by which the posterior membrane has an increased rate of MCC compared to the tracheal wall or exhibits a more robust MCC compared to the tracheal walls in *Bpifb1* KO mice. The fraction of total MCC due to posterior membrane transport versus that of the tracheal walls has also not been determined and will require further study. Clearly the tracheal posterior membrane can transport a substantial volume of mucus under conditions in which tracheal wall transport fails (see Figure 4 and Supplementary Videos 6, 7). This ability of the tracheal posterior membrane to transport particles is likely very dynamic and influenced by the contractile state of the trachealis muscle.

It has been well established that the quantity and composition of mucus lining the airways can have a profound effect on the rate of MCC, and in the mouse, MCC in both the upper and lower airways is very sensitive to alterations in the quantity and composition of the mucus (Button et al., 2012; Roy et al., 2014; Grubb et al., 2016; Hancock et al., 2018). Others have shown that as the mucus concentration increases, the rate of cilial transport is reduced (Button et al., 2012; Sears et al., 2015). In addition, we have found that a reduced level or complete absence of MUC5B results in a significant impairment of tracheal and nasal MCC (Roy et al., 2014; Grubb et al., 2016; Chen et al., 2018). Interestingly, in some mouse models (Chen et al., 2018), while a reduced (but not absent) level of MUC5B significantly reduced the rate of MCC in the lower airways, the rate of MCC in the nasal cavity was not affected (Chen et al., 2018). We (Rogers et al., 2018) and others (Hancock et al., 2018) have noted that an increased mass/concentration of MUC5B or an increased mass of particulate matter in the murine lower airways results in a marked decrease in tracheal MCC, yet the MCC in the nasal cavity was unaffected (Rogers et al., 2018).

In the PNP, the mucus layer (and thus presumably the blanket) had a significantly greater mass of sialic acid, a surrogate marker of mucins, compared to the trachea. This suggests a significantly greater volume of mucus in the PNP which aligns with our observations that the quantity of mucus is great enough in the PNP such that it can sometimes be plucked off with forceps. In addition, when the filter papers used in sialic acid collection were removed from the PNP, a string of mucus was often attached to the filter paper. In contrast, in the trachea, the epithelial surface appeared fairly dry, and mucus could never be removed with forceps, nor was it ever visually observed attached to the filter paper strips. The increased mucus mass in the PNP is likely derived from the contributions of the nasal glands (anterior), submucosal glands, Bowman’s glands (olfactory) and goblet cells, which are abundant in the nasal cavity but largely absent from the trachea. In contrast to rafts/streams of mucus, an extensive blanket of mucus would provide a greater surface for particle trapping which would be beneficial in the nasal cavity. However, it has been suggested that a blanket of mucus would be difficult to transport from the small distal airways having a larger surface area to the larger airways, having a much smaller surface area (Fakih et al., 2020). The murine distal airways appear to have over-come this problem by transporting mucus in rafts/streams of mucus rather than a mucus blanket.

In summary, MCC differs both qualitatively and quantitatively in the nose (posterior nasopharynx) compared to the trachea. In the nasopharynx, particles are transported rapidly on a continuous sheet of mucus, moving in a direct linear path to the distal end of the nasopharynx and then to the oropharynx where it is swallowed. In contrast, the tracheal walls, while covered by a thin sheet of mucus, appear to move particles on discontinuous patches or streams of mucus, often in indirect routes toward the tracheal posterior membrane or epiglottis. In the mouse, the tracheal posterior membrane (under the conditions in this study) moves mucus cephalad to the epiglottis in continuous streams likely in grooves created by the trachealis muscle, in a more rapid and linear path compared to the tracheal walls. In the nasal posterior nasopharynx, the greater cilial density, significantly higher CBF and greater mucus mass supplied by numerous glands contribute to achieve the more robust MCC in this region compared to that in the trachea. The robust mucus blanket in the PNP, not only plays a major role in trapping particles before they reach the lower airways, but it likely plays a major role in humidification of the inspired air and prevents desiccation of the underlying epithelial layer.

## Data Availability Statement

The raw data supporting the conclusions of this article will be made available by the authors, without undue reservation.

## Ethics Statement

The animal study was reviewed and approved by University of North Carolina Institutional Animal Care and Use Committee.

## Author Contributions

TR carried out all the studies and analyzed the videos. BB adapted aerosolization apparatus for mouse use, set up fluorescent imaging equipment, assisted with manuscript preparation. CE carried out the sialic acid analysis. SK generated th*e Bpifb1* KO mice and discussed the experimental results. LO and AL-B discussed the experimental results and contributed the manuscript writing. MG wrote the software for bead track analysis. BG designed the experiments, carried out the studies, analyzed the data, and wrote the manuscript. All authors contributed to the article and approved the submitted version.

## Conflict of Interest

The authors declare that the research was conducted in the absence of any commercial or financial relationships that could be construed as a potential conflict of interest.

## Publisher’s Note

All claims expressed in this article are solely those of the authors and do not necessarily represent those of their affiliated organizations, or those of the publisher, the editors and the reviewers. Any product that may be evaluated in this article, or claim that may be made by its manufacturer, is not guaranteed or endorsed by the publisher.
