# Supplementary material for: Regional Differences in Mucociliary Clearance in the Upper and Lower Airways
Source: Front Physiol. 2022 Mar 9;13:842592. doi: 10.3389/fphys.2022.842592 (PMC8959816; doi:10.3389/fphys.2022.842592)
Supplement: Supplementary file 1 [file Data_Sheet_1.DOCX]

Supplementary Material

# Supplementary Data

## Methods

To measure MCC in the rabbit trachea, the rabbit was given a pre- anesthetic dose of xylazine (5mg/kg i. m.), and then anesthetized with 3% isoflurane. Once on a surgical anesthetic plane, the rabbit was euthanized by aortic exsanguination. The upper trachea was surgically opened, and fluorescent beads were aerosolized directly onto the tracheal surface. Humidity and temperature were maintained as described for the mouse.

## Results

As demonstrated in the video (**Supplementary Video 5**), when beads were aerosolized on the open rabbit trachea, many of the beads on the walls did not move (as was often observed in the mouse) whereas beads deposited on the posterior membrane appeared as continuous streams flowing linearly on the posterior membrane. The beads on the walls near the posterior membrane tended to collect in streams of mucus and moved, in many cases non-linearly towards the posterior membrane, similar to what we observed in the mouse. In contrast, the beads on the posterior membrane moved as more uniform linear streams towards the epiglottis. As in the mouse, the rate of MCC on the posterior membrane of the rabbit trachea was significantly greater than that measured on the walls of the rabbit trachea (**Supplementary Figure 3**).

# Supplementary Video Legends

**Supplementary Video 1.** Mucociliary clearance of fluorescent beads in an intact mouse posterior nasopharynx. The beads, on the ventral epithelial layer tend to clump, but they appear to be transported at about the same rate regardless of size. It is likely that the clumping of beads occurred when they were introduced into the nasal cavity. Representative video from 10 mice.

**Supplementary Video 2.** Beads (3 µm) aerosolized on the opened posterior nasopharynx (ventral epithelial layer has been removed) in a mouse demonstrating that all beads are transported at about the same rate on the blanket of mucus covering the epithelial layer. Representative video from 5 mice.

**Supplementary Video 3.** Tracheal MCC viewed through the ventral wall of an intact tracheal. The fluorescent beads (200 nm) were delivered by nasal inhalation. Some beads seem to be stationary, and some beads seem to follow a stream/river of mucus. Representative video from 8 mice

**Supplementary Video 4.** Mucociliary transport on the posterior membrane of a murine trachea. Beads (200nm) were directly aerosolized on the open trachea just prior to starting the video. (Because it is difficult to visualize the posterior membrane, a significant portion of the ventral and lateral tracheal walls has been removed in this preparation). The posterior membrane is represented by the small stream of moving beads in the middle of the image. Very little vectorial transport of beads is seen on the walls of these preparations. Representative video from 8 mice.

**Supplementary Video 5.** MCC on the walls and the posterior membrane in the rabbit trachea. Because the rabbit trachea was so much larger than that of the mouse, we could better aerosolize the beads (200 nm) through a slit in the ventral wall of the trachea and view the posterior membrane. The cut edges of the cartilage (out of focus) in the ventral wall can be seen at the top edge of the video. The beads on the posterior membrane demarcated by the dashed yellow line, tend to flow in longitudinal rivers (rugae). (In this video the beads had been aerosolized several minutes before the video was started and thus the beads have already accumulated on the posterior membrane. (Representative video from 10 rabbits)

**Supplementary Video 6.** Tracheal (ventral wall) MCC in a WT mouse (control mouse for Bifb1 KO) first half of video. Second half of video, the trachea has been opened and MCC on the posterior membrane is visible. Representative video from 5 mice.

**Supplementary Video 7.** Tracheal (ventral wall) MCC in a Bpifb1 KO mouse, first part of video. Second part of video, the trachea has been opened and MCC on the posterior membrane is visible. There appears to be a much wider area of beads on the KO posterior membrane, but we have not rigorously determined if this is the case. Representative video from 5 mice.

**Supplementary Video 8.** Transport of the blanket of mucus stretched across the ventral region of the PNP devoid of epithelial cells. It is clear that this blanket is moving as it is pulled across the breach by the surrounding cilia. Towards the end of the video, the mucus blanket can be seen to spontaneously rupture (likely due to desiccation as this preparation was not humidified) and the beating cilia are visible on the dorsal surface of the PNP. Representative video from 5 mice.

**Supplementary Video 9.** In the PNP, as the mucus blanket was transported across the breached area, we aerosolized 3µm beads on the outside of this blanket, again demonstrating that the blanket was transported across the region devoid of ciliated epithelia. (Compare to Video #1 where beads were transported on the luminal side of the blanket in the intact PNP.) Representative video from 5 mice.

**Supplementary Video 10.** Tracheal “mucus” blanket exposed by dissecting away connective and epithelial tissue between the cartilaginous ring in a murine trachea. Beads were then aerosolized on the outside of the trachea and can be seen to deposit on the basolateral side of the mucus blanket. The mucus blanket stretching across the breached epithelial region never exhibited vectorial transport although some of the beads moved around slightly. At the end of the video, the tracheal mucus blanket was ruptured by the insertion of a pipette. (Representative video from 6 mice).

## Supplementary Figures


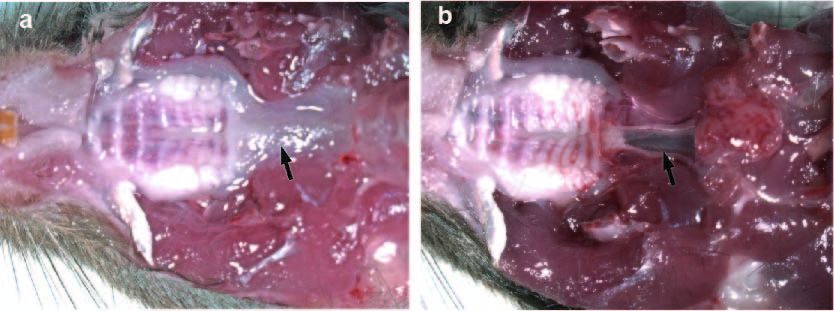


**Supplementary Figure 1.** (**A**) En Face view of the murine hard palate (anterior nasopharynx)/soft palate (posterior nasopharynx) region after the lower jaw has been removed. Arrow indicates squamous layer covering the posterior nasopharynx. (**B**) En Face view of the murine hard palate/soft palate. Arrow indicates the PNP after squamous layer has been removed, exposing the basolateral side of the ciliated epithelia lining the PNP. The ciliated epithelia is intact in this preparation and this intact epithelial layer provides the window through which we MCC and CBF.


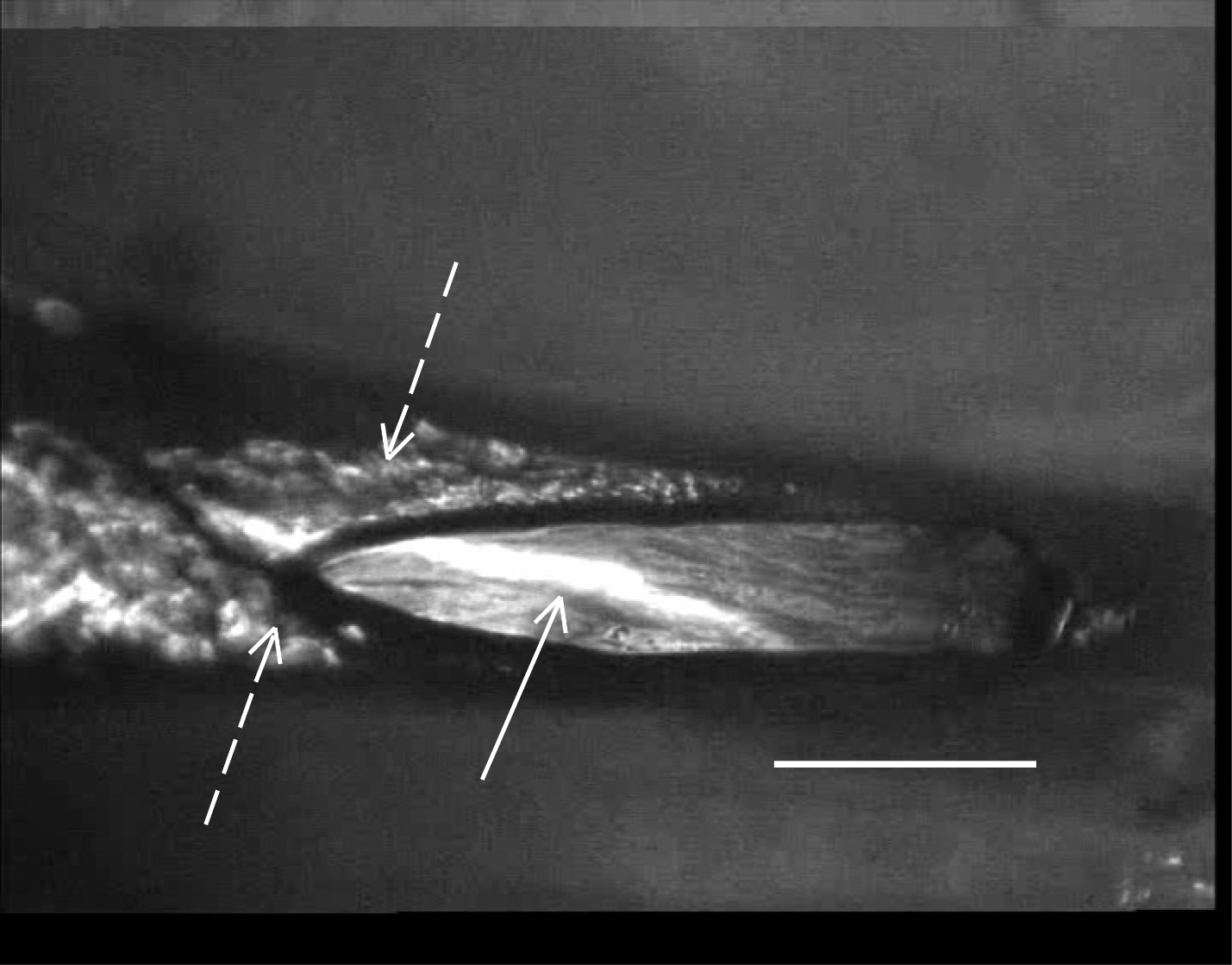


**Supplementary Figure 2.** Image of posterior nasopharynx taken under UV light after a region of the ciliated epithelium was breached exposing a blanket of mucus stretched across the breached region. The mucus appeared to have an oil sheen. Dashed arrows are on the basolateral side of the intact ciliated layer. Solid arrow is the mucus blanket across the region in which the ciliated epithelia has been breached.

Size bar = 500μm


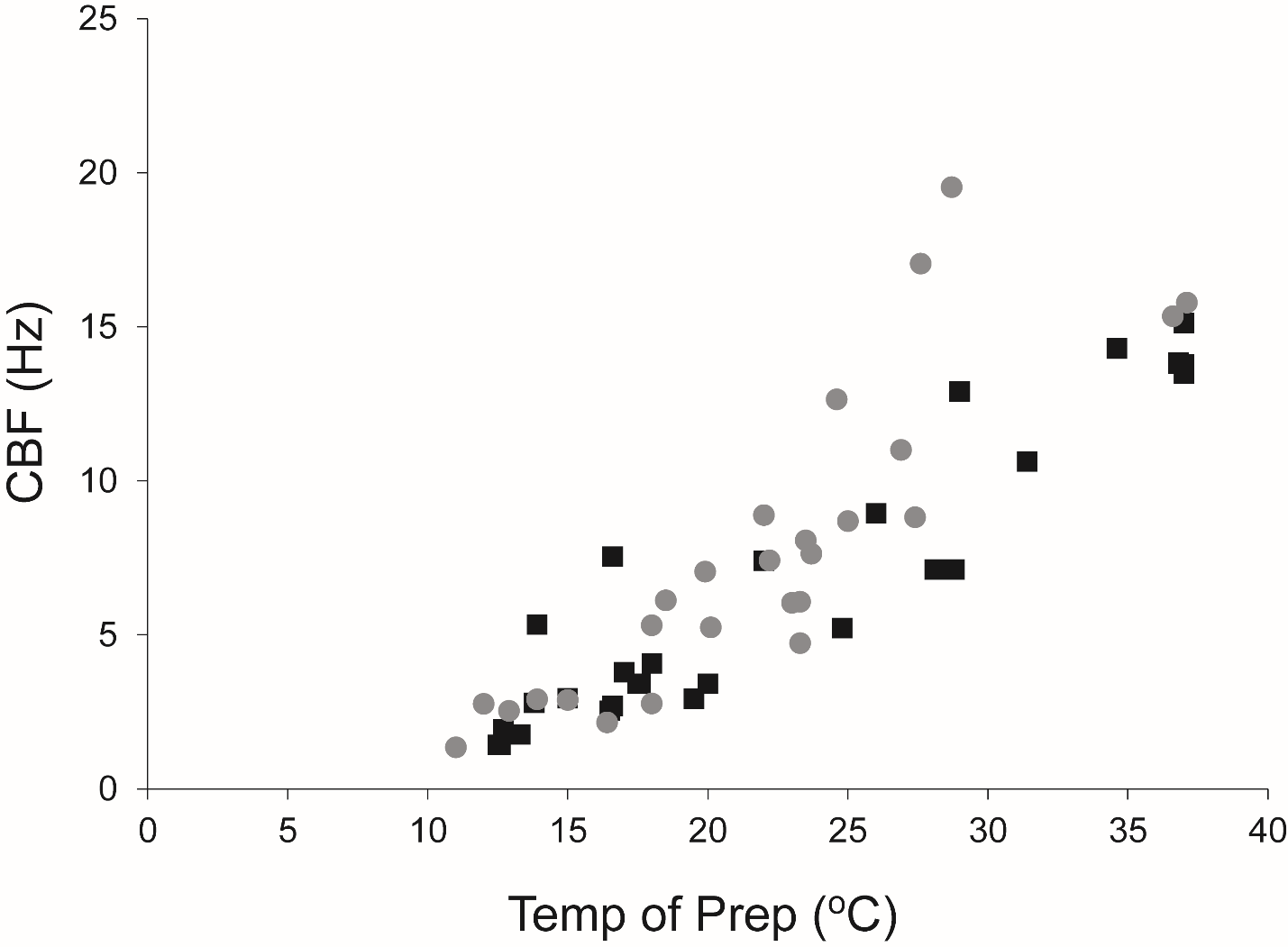


**Supplementary Figure 3.** Effect of temperature on CBF in the trachea and Nasopharynx. Grey circles are data from intact trachea and solid squares are the data from the PNP (n=2 mice PNP, N=3 trachea).


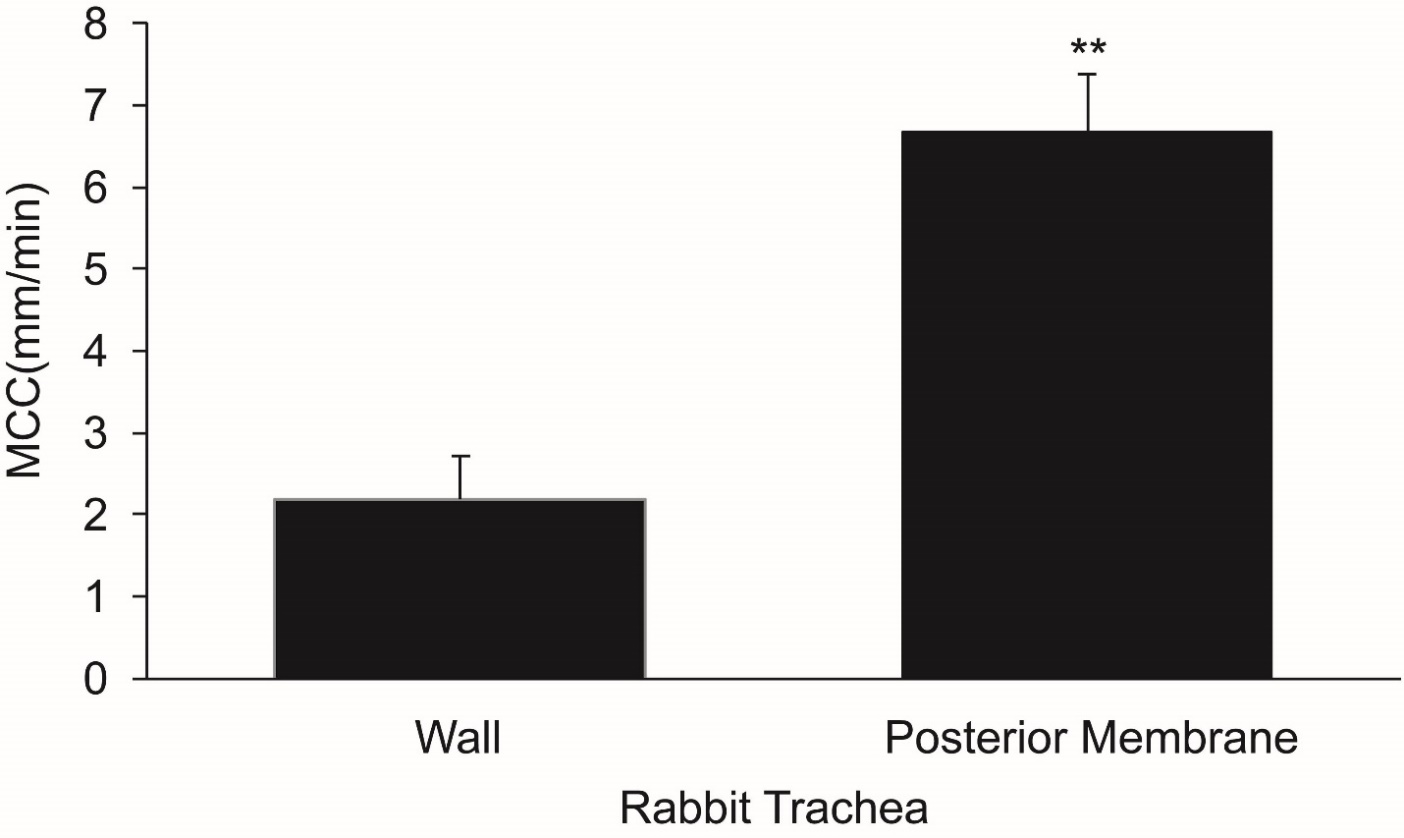


**Supplementary Figure 4.** MCC on the rabbit tracheal wall compared to the tracheal posterior membrane. N=5 rabbits for tracheal wall and n=10 for posterior membrane. ** p ≤ 0.001 tracheal wall vs tracheal posterior membrane.
