# Supplementary figures and images for: Regional Differences in Mucociliary Clearance in the Upper and Lower Airways
Source: Front Physiol. 2022 Mar 9;13:842592. doi: 10.3389/fphys.2022.842592 (PMC8959816; doi:10.3389/fphys.2022.842592)

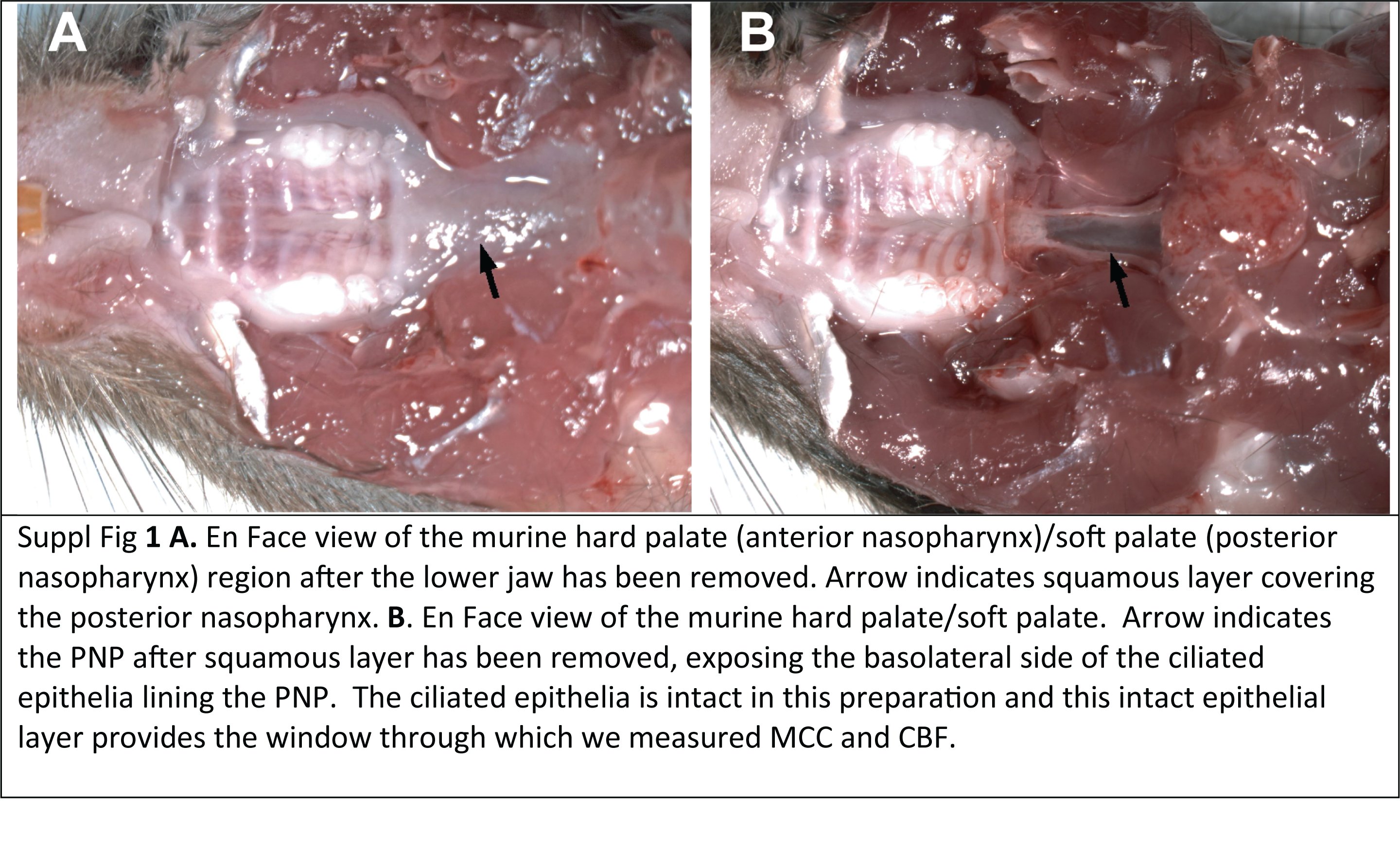

Supplement: Supplementary file 2 [file Image_1.jpg]

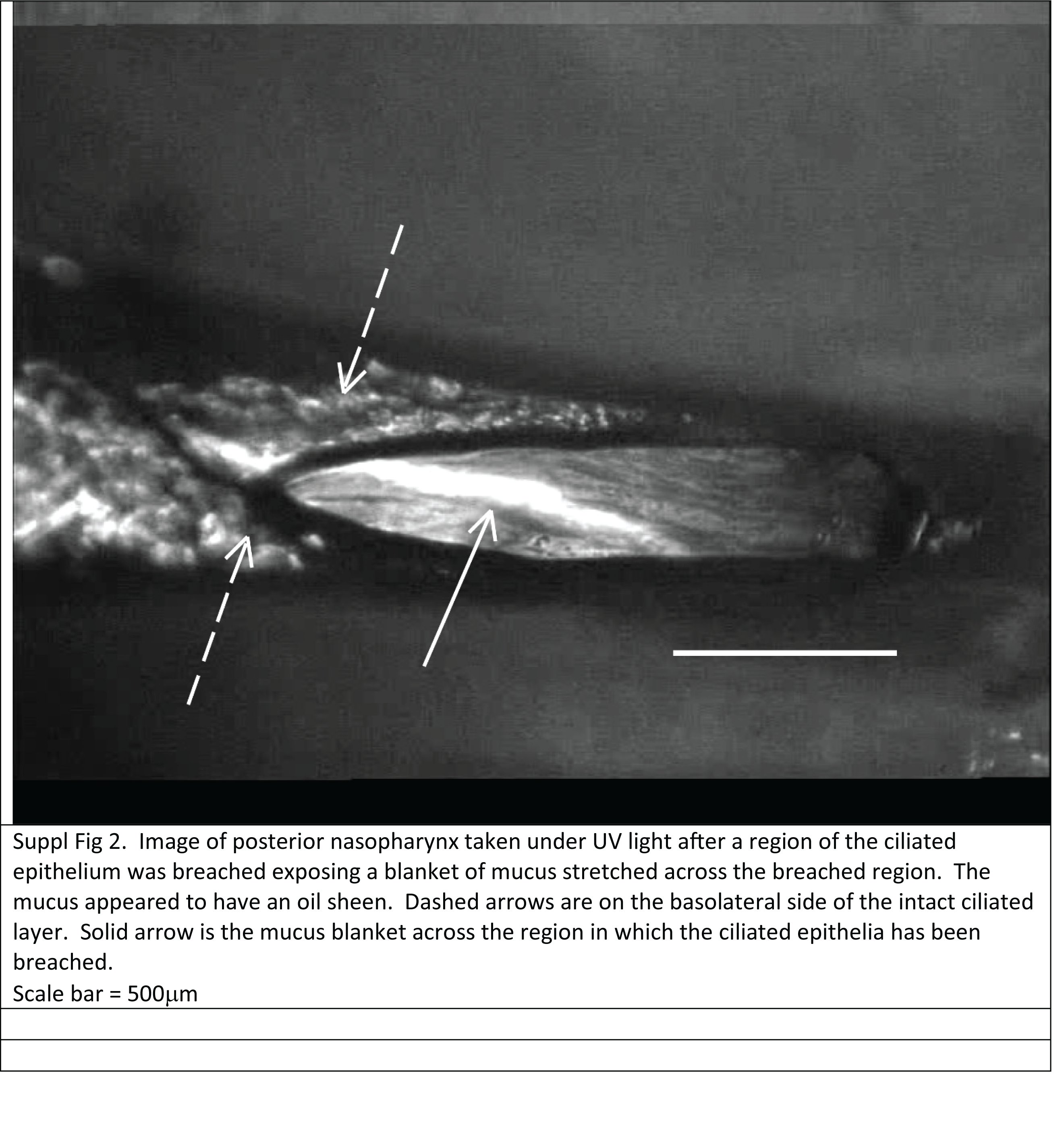

Supplement: Supplementary file 3 [file Image_2.jpg]

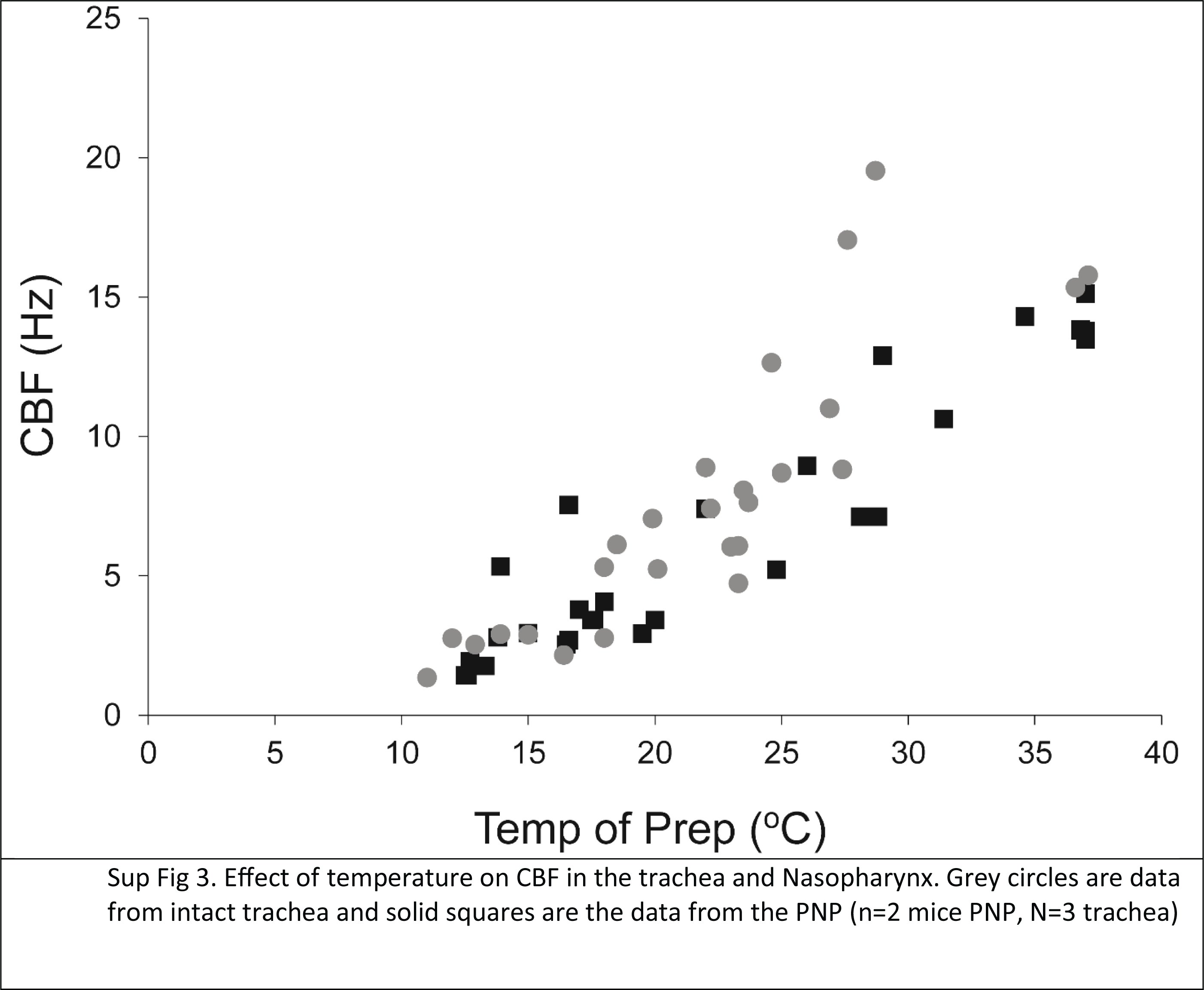

Supplement: Supplementary file 4 [file Image_3.jpg]

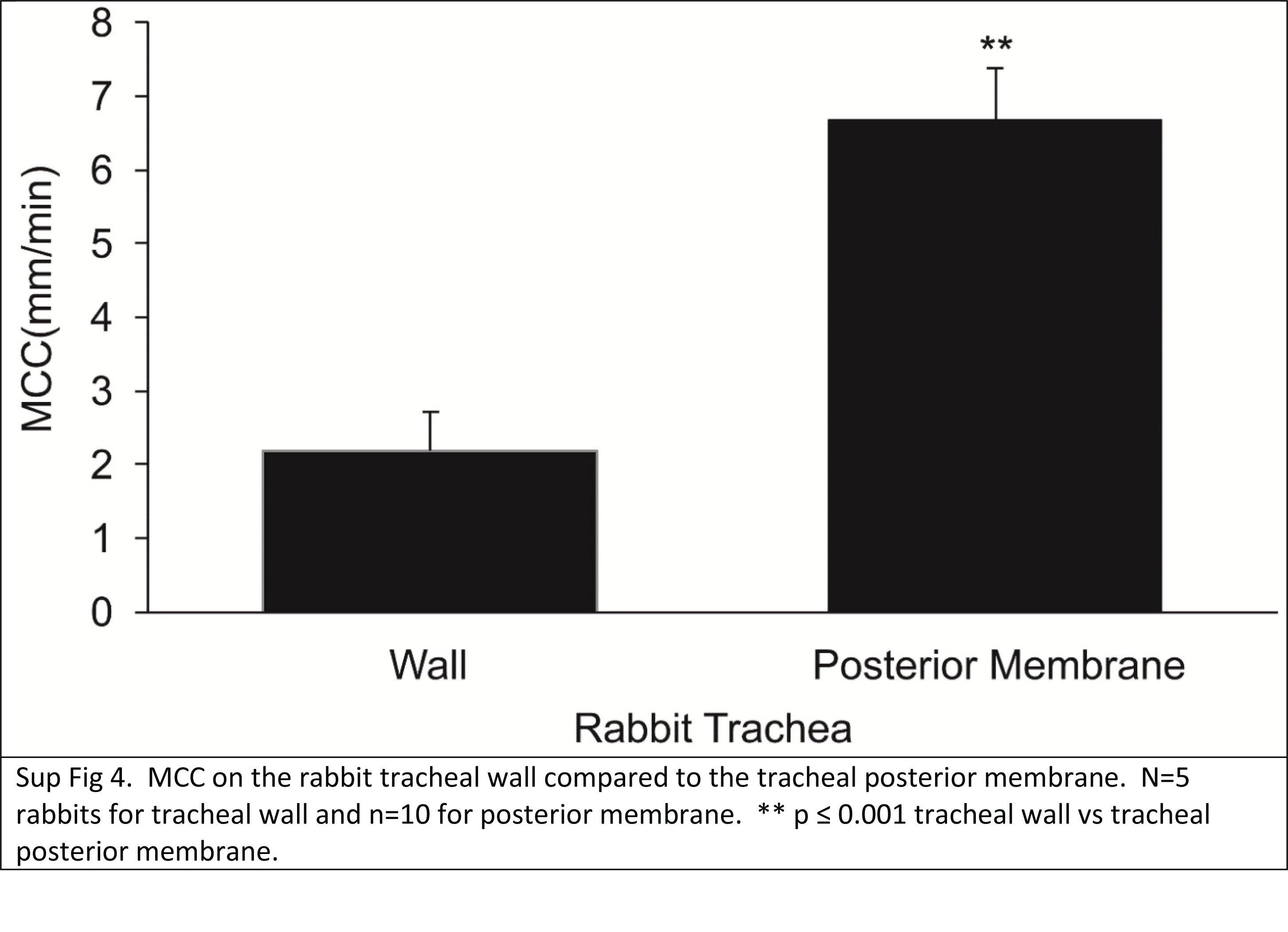

Supplement: Supplementary file 5 [file Image_4.jpg]
